# Aquatic Turning Performance in Juvenile Loggerhead and Green Sea Turtles

**DOI:** 10.1093/iob/obag017

**Published:** 2026-04-24

**Authors:** M T Wileyto, F E Fish, S E Trail, J Wyneken, R Kramer-Bottiglio

**Affiliations:** Department of Biology, West Chester University, West Chester, PA 19383, USA; Department of Biology, West Chester University, West Chester, PA 19383, USA; Department of Biological Sciences, Florida Atlantic University, Boca Rotan, FL 33431-0991, USA; Department of Biological Sciences, Florida Atlantic University, Boca Rotan, FL 33431-0991, USA; School of Engineering & Applied Science, Yale University, New Haven, CT 06520, USA

## Abstract

Sea turtles are known for being accomplished swimmers, capable of covering great distances. Despite the importance of maneuverability, little has been done to define the mechanics of turning in these marine organisms. To examine turning performance, juvenile loggerhead (*Caretta caretta*) and green sea turtles (*Chelonia mydas*) were video recorded overhead in pursuit of food in a large tank. The turtles were observed performing two types of yawing turns: pure rotational turns and translational turns. The trajectory of points on the body of the turtles (rostrum, anterior of shell, and posterior of the shell) were digitally tracked. X and Y coordinates were analyzed over time with a custom MatLab code, which estimated center of mass (COM), turn radius, and turn rate (angular velocity). Turning performance significantly differed between sea turtle species during rotational turns but did not significantly differ between sea turtle species during translational turns. Asymmetries were observed in limb usage during turns, which affected the angular velocity. Stroke frequencies significantly differ between turn type, but not between species. Analysis of the coordination of flippers suggests that *C. caretta* more often initiates turns with both forelimbs, while *C. mydas* more often initiates turns with one forelimb. When mapped categorically with other swimming taxa, sea turtles, resembled most other rigid-bodied swimmers, and placed below the guideline separating flexible-bodied swimmers and rigid-bodied swimmers. These data support the hypothesis that having a rigid body-plan constrains turning performance.

## Resúmen

Es conocido que las tortugas marinas son nadadoras eficientes, capaces de cubrir grandes distancias. A pesar de la importancia de la maniobrabilidad, existed pocos estudios sobre la mecánica de los giros en estos organismos marinos. Para examinar el rendimiento de los giros, se grabaron en video desde arriba a tortugas caguamas juveniles (*Caretta caretta*) y tortugas verdes (*Chelonia mydas*) mientras perseguían comida en un tanque grande. Se observó a las tortugas realizando dos tipos de giros oscilantes en el eje vertical: giros puramente rotacionales y giros traslacionales. La trayectoria de puntos en el cuerpo de las tortugas (rostro, parte anterior del caparazón y parte posterior del caparazón) se rastreó digitalmente. Se analizaron las coordenadas X y Y como función del tiempo utilizando un código especializado de MatLab, para estimar el centro de masa (COM), el radio de giro y la velocidad de giro (velocidad angular). El rendimiento de los giros difirió significativamente entre las dos especies estudiadas durante los giros rotacionales, pero no difirió significativamente entre las especies estudiadas durante los giros traslacionales. Se observaron asimetrías observado en el uso de las extremidades durante los giros, lo que afectó la velocidad angular. Las frecuencias de brazada difirieron significativamente entre el tipo de giro, pero no entre especies. El análisis de la coordinación de las aletas sugiere que *C. caretta* inicia los giros con mayor frecuencia usando ambas extremidades anteriores, mientras que *C. mydas* inicia los giros con mayor frecuencia usando una sola extremidad anterior. Cuando se compara categóricamente con otros taxones de animals nadadores, las tortugas marinas se asemejaron a la mayoría de los nadadores de cuerpo rígido, y se ubicaron por debajo de la guía que separa a los nadadores de cuerpo flexible de los nadadores de cuerpo rígido. Estos datos apoyan la hipótesis de que tener un plan corporal rígido limita el desempeño en los giros.

## Introduction

**Fig. 1:**
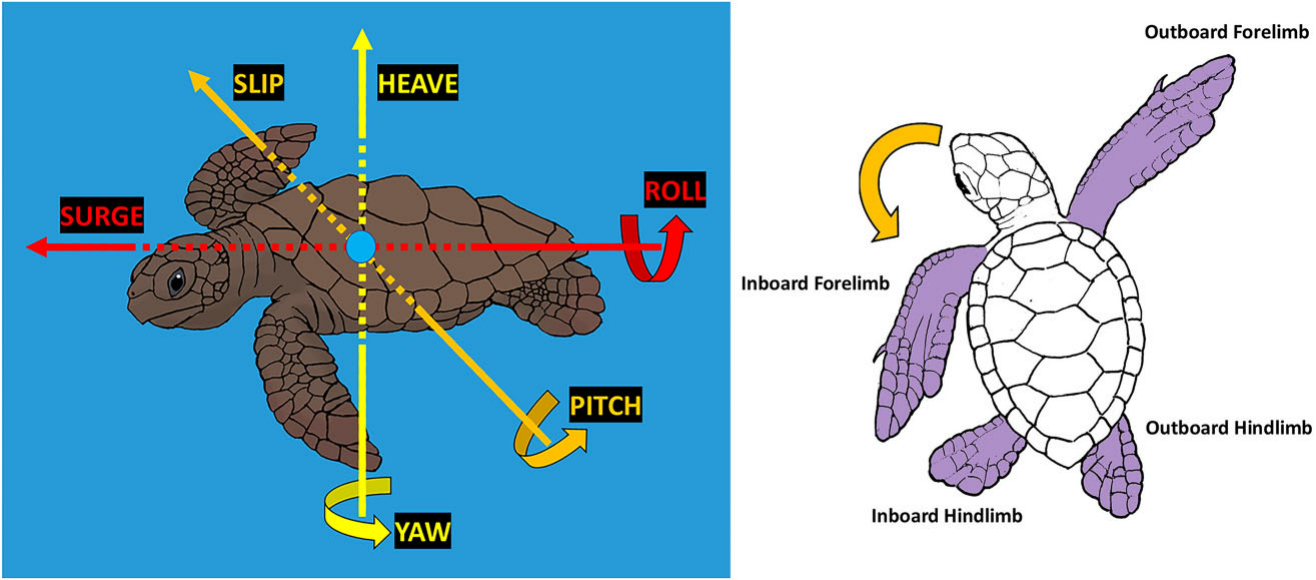
Orientation terminology. Axes of movement and degree of freedom (left). Pure rotational movement in the study consists of only yaw. Translational movement in the study consists of the combination of yaw and surge. Illustration of outboard and inboard limbs relative to turn direction (right).

For animals that live in complex environments and frequently encounter predators, turning performance is critical for navigation, evasion, and pursuit (Howland 1974; Fish 2002; Rivera et al. 2006; Parson et al. 2011). Within the three-dimensional space of the aquatic realm, an aquatic animal has six degrees of freedom around three orthogonal axes encompassing three translational (surge, heave, slip) and three rotational (roll, yaw, pitch) movements (Fig. 1). Turning performance with respect to these movements can be measured as the speed of a turn measured from the angular velocity (agility) and radius of the turn. (maneuverability) (Walker 2000; Rivera et al. 2006). There are two forms of turning: pure rotational turns (i.e., turn radius = 0), and translational turns (Rivera et al. 2006). Pure rotational turns, or reorientation involve only rotation about a vertical axis (yaw) through the center of mass of an animal (Fig. 1). Translational turns involve rotation about the yaw axis, while the animal is displaced along a curved trajectory, with a pathway that provides a measurable radius of the turn (Rivera et al. 2006).

Among aquatic animals, flexible-bodied animals are thought to have enhanced turning performance with respect to yaw (Fish 1999, 2002; Walker 2000; Walter and Carrier 2002; Rivera et al. 2006). The flexibility of the body makes these animals capable of turning in a circular space of less than half their body length, and it decreases their rotational inertia (Fish 1999, 2002; Walker 2000; Walter and Carrier 2002; Rivera et al. 2006). However, rigid-bodied animals, which are rendered inflexible by their own endoskeleton or exoskeleton, shell or other forms of body armor (e.g., batoids rays, boxfish, cuttlefish, squids, turtles; whirligig beetle) have a reduced turning performance (Foyle and O’Dor 1988; Fish 1999; Walker 2000; Fish and Niacastro 2003; Rivera et al. 2006, 2010; Parson et al. 2011; Webb 2011; Jastrebsky et al. 2016; Mayerl et al. 2017). Even though constrained, turning performance is possible by the use of multiple propulsive control surfaces (Walker 2000). One pattern that stands out is a performance tradeoff in the type of turn such that an animal may excel at agility or at maneuverability, or they do adequately well at both, but not excel. Examples of specialization in turn type include whirligig beetles (Fish and Niacastro 2003), which demonstrated high agility but low maneuverability (Fish 1999; Walker 2000), and boxfish, which demonstrated high maneuverability but low agility (Walker 2000).

Turtles are an ideal group for examining turning performance in rigid-bodied animals. The turtle carapace, which consists of ankylosed vertebrae, ribs (pleural bones), nuchal bone, peripherals and epidermal plates, restricts flexibility of the body posterior of the neck (Burke 1989; Rivera et al. 2006). Furthermore, all thrust produced comes from their pectoral and pelvic pairs of limbs (Zug 1971; Wyneken 1996; Rivera et al. 2006, 2011; Blob et al. 2007; Chikere et al. 2025). Numerous kinematic studies have been conducted on both freshwater and marine turtles (Davenport and Clough 1986; Renous and Bels 1991, 1993; Lohmann et al. 1995; Wyneken 1996; Rivera et al. 2006, 2011, 2013; Rivera and Blob 2010, 2013; Mayerl et al. 2017, 2019). Studies of turtle turning performance predominantly were conducted on freshwater turtles (Rivera et al. 2006; Mayerl et al. 2017). For turtles, the inboard (Fig. 1; limb directed toward the center of the turn) limbs of the turn are used as rudders or brakes, remaining at a stationary angle throughout the duration of a turn, while the outboard (Fig. 1; limb directed toward the outside of the turn) limbs maintain an asynchronous rowing motion (Mayerl et al. 2017). For juvenile painted turtles (*Chrysemys picta*), turning performance was intermediate between turning capabilities by boxfish and whirligig beetles, instead of excelling in either maneuvering or agility (Rivera et al. 2006). Adult freshwater turtles demonstrate high maneuverability but low agility comparable to other rigid-bodied taxa (Mayerl et al. 2019). Freshwater turtles use anteroposterior drag-based rowing motions for propulsion (Rivera et al. 2011, 2013; Rivera and Blob 2013), whereas sea turtles (superfamily Chelonioidea) use dorsoventral flapping motion of modified wing-like pectoral forelimbs for a lift-based propulsion (Pettigrew 1893; Wyneken 1996; Rivera et al. 2011). This difference in the structure and kinematics of the propulsive limbs could indicate differences in turning performance between freshwater and marine turtles.

While aquatic turning in sea turtles has been observed (Renous and Bels 1991; Lohmann et al. 1995; Avens et al. 2003), to date, no study has yet measured and compared the turning performance of multiple sea turtle species. Qualitative descriptions for *C. caretta* (loggerhead turtle), *C. mydas* (green turtle), and *Dermochelys coriacea* (leatherback turtle) reported turns results from asynchronous limb movement (Renous and Bels 1993; Wyneken 1996). In addition, turns in hatchling *C. caretta* involve a combination of forelimb propulsion and hindlimb rudder action (Davenport and Clough 1986; Lohmann et al. 1995; Wyneken 1996; Avens et al. 2003).

The goals of the present research are to understand the mechanisms of aquatic turning by sea turtles providing (1) a quantitative comparison between two species of sea turtles and (2) an assessment of flipper movements in relation to turning performance. It is predicted that *C. mydas* juveniles will have a higher turning performance than *C. caretta*, due to morphological differences between the two species, including the ratio of flipper length to shell length, dorsally keeled ridges on the carapace of *C. caretta* (Dougherty et al. 2010; Pereira et al. 2011). Additionally, it is predicted that turning performance is constrained in sea turtles because of their rigid body-plan.

## Materials and methods

### Experimental animals

**Fig. 2:**
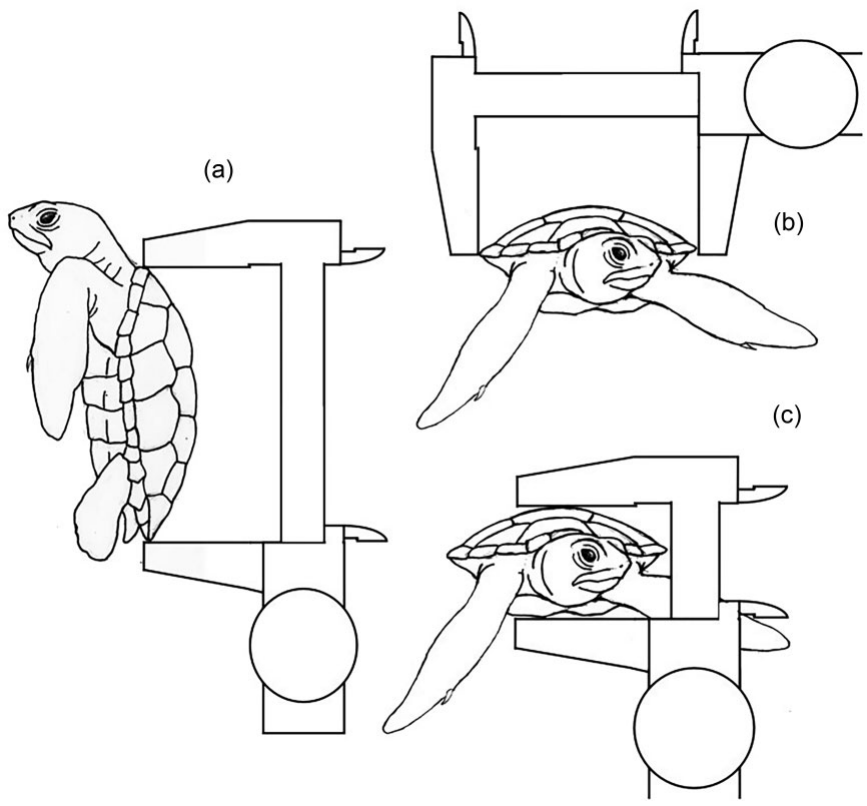
Standard measurements taken for turtle dimensions using dial calipers, including (a) carapace length, (b) carapace width, and (c) carapace depth.

Turtles were studied at Florida Atlantic University Marine Lab, Boca Raton, Florida (FWC permit MTP23-073 to JW). The experiments were approved by the West Chester University Institutional Animals Care and Use Committee (IACUC) Protocol #202,001. Ten young juvenile turtles: *C. caretta* (n = 5) and *C. mydas* (n = 5) were recorded during swimming. The two species examined are both related within the family Cheloniidae (Pritchard 2017), although they are in different subfamilies which diverged about 63 MYA (Naro-Maciel et al. 2008). Videos were not used where discreet points on the turtles were difficult to track as the limbs were obscured due to splashing and turns were not executed. Videos on only 6 turtles (three *C. caretta* and three *C. mydas*) could be analyzed that executed turns. These species were used as they were available in the laboratory of Jeanette Wyneken. The turtles were approximately 2 months old. Turtles’ straight carapace lengths, carapace widths, and maximum carapace depths were measured using dial calipers (Fig. 2) and mass was measured to the nearest 0.1 g with a calibrated digital balance. Body morphometrics of the individual sea turtles are provided in Table 1. The forelimbs are the elongated, flattened manus and antebrachium of the forelimbs. The paddle-like pes and crus describe the hindlimbs. Forelimb lengths were measured using Tracker (Douglas Brown, ver. 5.0.7), and averaged per species, with the mean forelimb length of *C. caretta* being 5.45 cm, and the mean forelimb length of *C. mydas* being 5.65 cm. Forelimb length was then divided by the average carapace length of each species.

**Table 1:** Body measurements of the sea turtles that provided data, Caretta caretta (CC) and Chelonia mydas (CM)

Turtle ID	Carapace Length (m)	Carapace Width (m)	Carapace Depth (m)	Mass (g)	Flipper-Shell Length
CC21-1501	0.1026	0.0941	0.0484	208	0.5312
CC21-1504	0.1121	0.0995	0.0522	253.6	0.4862
CC21-1505	0.1038	0.0896	0.0474	195.4	0.5251
CM21-1304	0.0959	0.0872	0.0389	143.6	0.5892
CM21-1307	0.0932	0.0851	0.0379	128.6	0.6062
CM21-1309	0.0967	0.0824	0.0416	143.4	0.5843

### Video recording and analysis

**Fig. 3:**
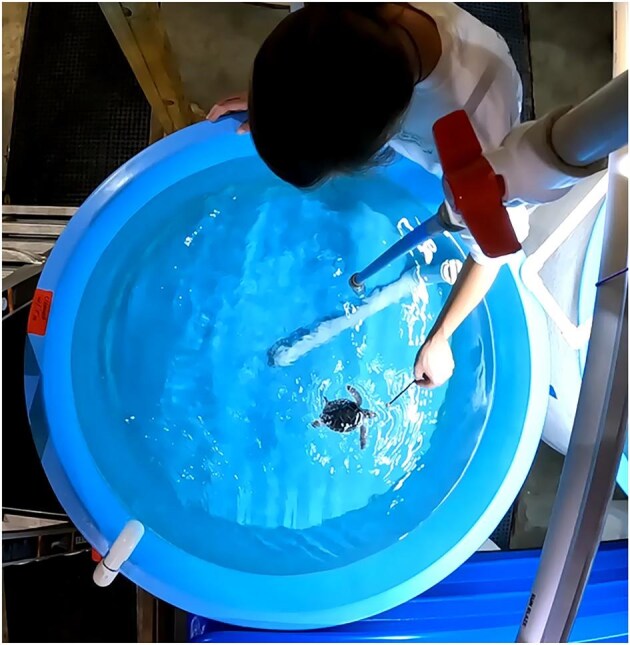
Top view of the experimental tank filled with salt water. Inside, a green sea turtle (*Chelonia mydas*) neonate investigates food on the end of a wand.

To record data on turning performance in a horizontal plane, a GoPro10 camera (GoPro, Inc.) was placed 1 m above a circular tub 1.14 m in diameter, with a water depth of 0.42 m, providing a dorsal view of the turtles (Fig. 3). Turtles were recorded in the center of the field of view to reduce distortion of the GoPro lens. Turtles were recorded at 30 frames/s. Prior to testing, a ruler was placed at the water’s surface to scale the videos. Each turtle was observed and video recorded singly in the water. The turtle was encouraged to turn around the tub by providing individual food cubes on the end of a flexible wand (Fig. 3)—a normal husbandry process for these turtles. During the simulated pursuit, the food-wand motions included a circular arc around the periphery of the tub, or were provided closer to the turtle in circular motions. Each turtle also was recorded turning on its own without food inducement while exploring its surroundings. All turning movements of the turtles were confined to the surface of the water.

**Fig. 4:**
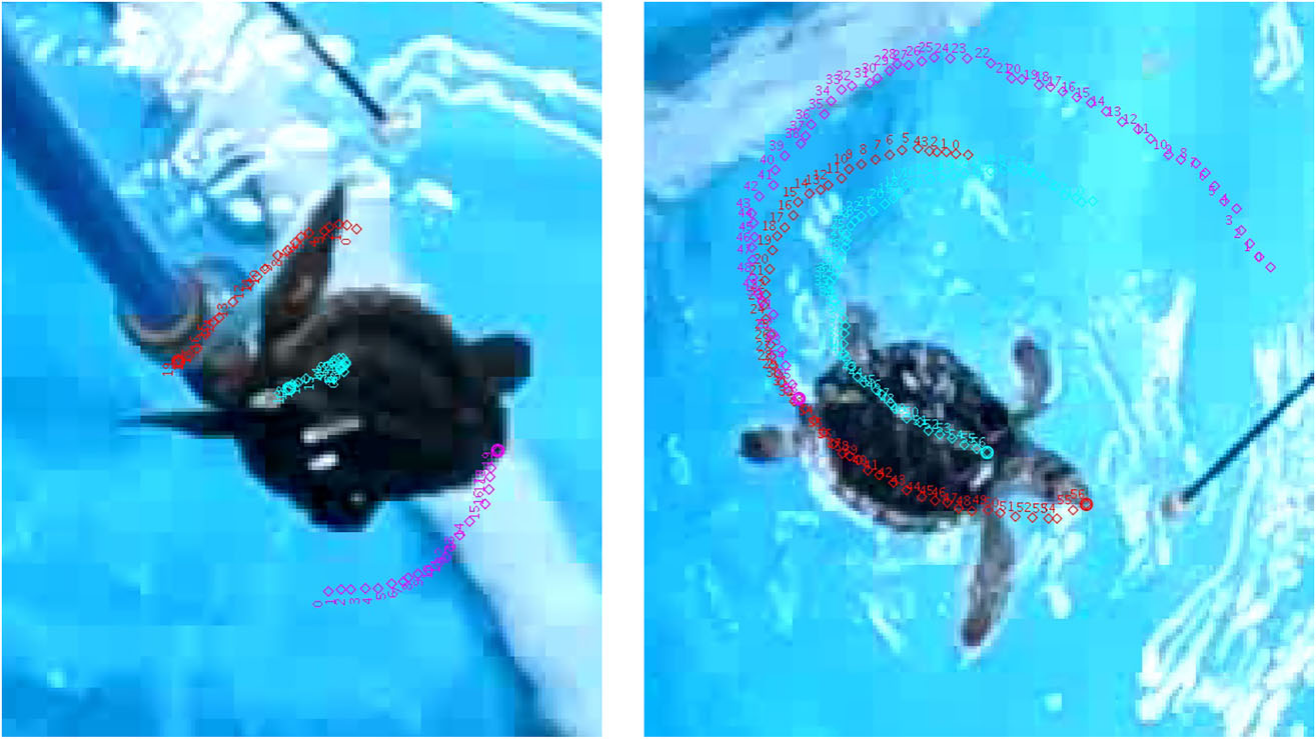
Images from video of sea turtle turning. Symbols show the position of the rostrum (red), anterior of carapace (blue), and posterior of the carapace (purple) for a rotational turn (left) and translational turn (right).

Movements of the turtles from the scaled videos were sliced into short clips that only showed the turtles turning. Incomplete turns were eliminated from the study. The clips, each of which served as a trial, were then converted from MP4 files to AVI files using Adapter (Macroplant LLC, ver. 1.0) software for analysis in Tracker (Douglas Brown, ver. 5.0.7). Three defined points on the turtles were tracked, including the rostrum, the anterior of the carapace, and the posterior of the carapace (Fig. 4). Points digitally placed on the turtles throughout the duration of the turns provided X and Y coordinate pairs that were analyzed using a custom MatLab code (Mathworks, Inc, ver. R2022a).

The MatLab code calculated a virtual center of mass (COM). The COM was based on measurements of turtle specimens from the herpetology collection at the Academy of Natural Sciences of Drexel University. Sea turtle specimens included one medium-sized juvenile loggerhead sea turtle (*C. caretta*), and five hatchling leatherbacks sea turtles (*D. coriacea*). The COM was determined by balancing the specimens on a metal bar, and measuring the distance on the body from the front of the carapace to the COM. The virtual COM as an approximation of the position of COM was used to determine the turn radius and turn rate of the turtles.

In addition to points placed on the body, marker dots were tracked for the tips of forelimbs and hindlimbs to assess variables of their strokes during a turn, as well as make observations on which limbs were used for the turns. Forelimbs and hindlimbs were identified based on their position as outboard or inboard and categorized as outboard forelimb (OBFF), inboard forelimb (IBFF), outboard hindlimb (OBHF), and inboard hindlimb (IBHF). Strokes performed by these limbs in each turn were measured for their arc chord (cm) and duration (s). These variables were used to examine stroke speed and stroke frequency.

### Kinematic turning variables

There were four primary variables used for analysis. Three of these were continuous variables: angular velocity (deg s^−1^), stroke speed (m s^−1^), and stroke frequency (Hz). Angular velocity was obtained from the COM point calculated in MatLab and was used to measure turning performance. Stroke speed was measured as arc chord length divided by the duration of the stroke. Stroke frequency was measured as the number of strokes divided by the time. Limb usage (in-use vs. not in-use) was a binary variable. Limb usage is based on observation of whether or not a flipper or foot was used.

### Statistical analysis

All analyses were conducted using STATA (StataCorp LLC, version 18.0), with *p* < 0.05 as the criterion for significance. Variation about means was expressed as ± 1 standard deviation. Shapiro–Wilk tests were used to determine whether data were normally distributed, with a focus on a V-value (which is not sample size dependent). The V- measure is 1.0 for normal data, with large V-values indicating a severe departure from normality. For variables found to be non-normal, bootstrapping methods were used to generate exact confidence intervals, exact p-values, and to assess the impact of non-normal data on our linear mixed-model estimates. Bootstrap sampled random clusters with replacement, assigning new cluster ID’s for each analysis replicate. If the impact was large (V > 5), the bootstrap-generated data were used; if not (V < 5), the original data were used.

Turning outcomes were repeated measures and were clustered based on individual or individual by turn. Turning radii for both species were analyzed using linear regressions. Angular velocity, stroke speed and flipper stroke frequency were analyzed using linear mixed models regression. Mixed models correct for repeated measures by separately estimating pure error variance and variation among individuals and turns (random effects). For angular velocity, rotational and translational turns were sorted, and the comparison was made by species. Models involving flipper speed included categorical or binary predictor terms for limb (4 categories: IBFF, IBHF, OBFF, OBHF), species (*C. caretta, C. mydas*), and turn type (rotational, translational). For each outcome, the analysis began with the full 3-way interaction model, and removed non-significant interactions hierarchically, starting with the 3-way interaction term.

A contingency table and correlation analyses were conducted to examine limb usage, looking at both correlation between two limbs, and the significance of that correlation. The pair of flippers, in this case, was any pairing of flippers (i.e., outboard foreflipper and inboard hindlimb, inboard foreflipper and inboard hindlimb, outboard foreflipper and inboard foreflipper). Z-tests were constructed to identify differences in limb-pair usage between species if a given pair (1) shows significance in one species, and (2) the correlations of one species mismatch that of the other species.

## Results

### Specimens

A total of 201 trials were analyzed for the angular velocity of turns. Of those, 85 trials were from *C. caretta* (n = 3), and 116 trials were from *C. mydas* (n = 3). The *C. caretta* trials consisted of 37 rotational turns, and 48 translational turns. Rotational turns were defined as yawing turns in which the turtle would rotate about its center of mass, and translational turns were defined as a combination of yawing and surging so that the turtle moved along an arc (Fig. 4; Movie 1, 2). The *C. mydas* trials consisted of 78 rotational turns, and 38 translational turns. All six turtles performed clockwise and counterclockwise turns. Often, turns were initiated by the head, which would tilt in the direction of the turn.

Of the 201 trials, 104 trials provided limb movement results; 54 observations came from *C. caretta* (n = 2) as 32 rotational turns and 22 translational turns. *C. mydas* (n = 3) provided 50 trials, which consisted of 38 rotational turns and 12 translational turns. While in 3 of the 38 rotational turns *C. mydas* exclusively stroked with the inboard hindlimb, *C. caretta* had 9 turns, including 8 rotational, and 1 translational, that were performed exclusively with the hindlimbs.

### Angular velocity of sea turtles

**Fig. 5:**
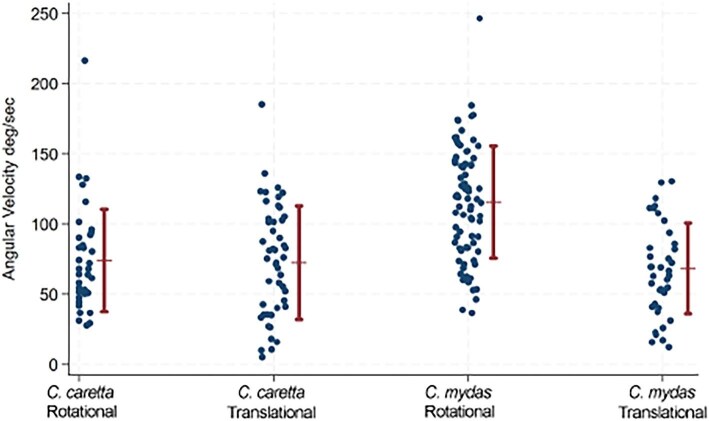
Means of angular velocity (deg s^−1^) (red) over raw angular velocity data (blue).

Turning performances for both species were investigated for differences using linear mixed model regressions. Angular velocity was distributed normally (V = 2.789), and therefore kept. An interaction (species and turn type) was tested, but the interaction was not significant, and therefore not included. To see original interaction outcome, see S3. Mean turning performances for each species are measured against the raw data in Fig. 5.

For *C. caretta*, the angular velocities of rotational turns did not differ from the angular velocities of translational turns (β=−9.89; CI=(−30.79, 11.01); z=−0.93; *p* = 0.354); whereas for *C. mydas*, the angular velocities of rotational and translational turns differed significantly (β=−28.03; CI=(−54.84, −1.21); z=−2.05; *p* = 0.040).

**Fig. 6:**
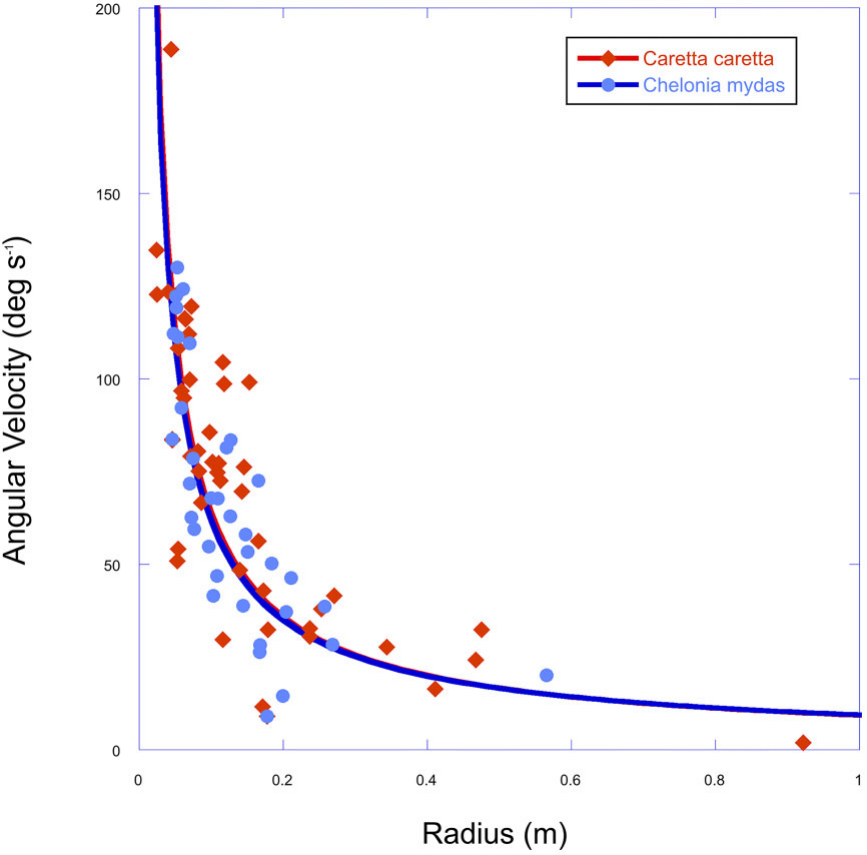
Relationship between angular velocity and turning radius for sea turtles; *Caretta caretta* (red diamonds; n = 3) and *Chelonia mydas* (blue circles; n = 3). Note that turning radius decreases with increasing angular velocity.

There was considerable variation in turning radius for both species. However, the turning radii between the two species did not differ significantly (β=−1.82; CI=(−12.89, 9.25); t(5)=−0.42; p = 0.690). During translational turns, there was an inverse relationship between the angular velocity of the turn, and the radius of the turn (Fig. 6).

### Use of limbs for turning

**Fig. 7:**
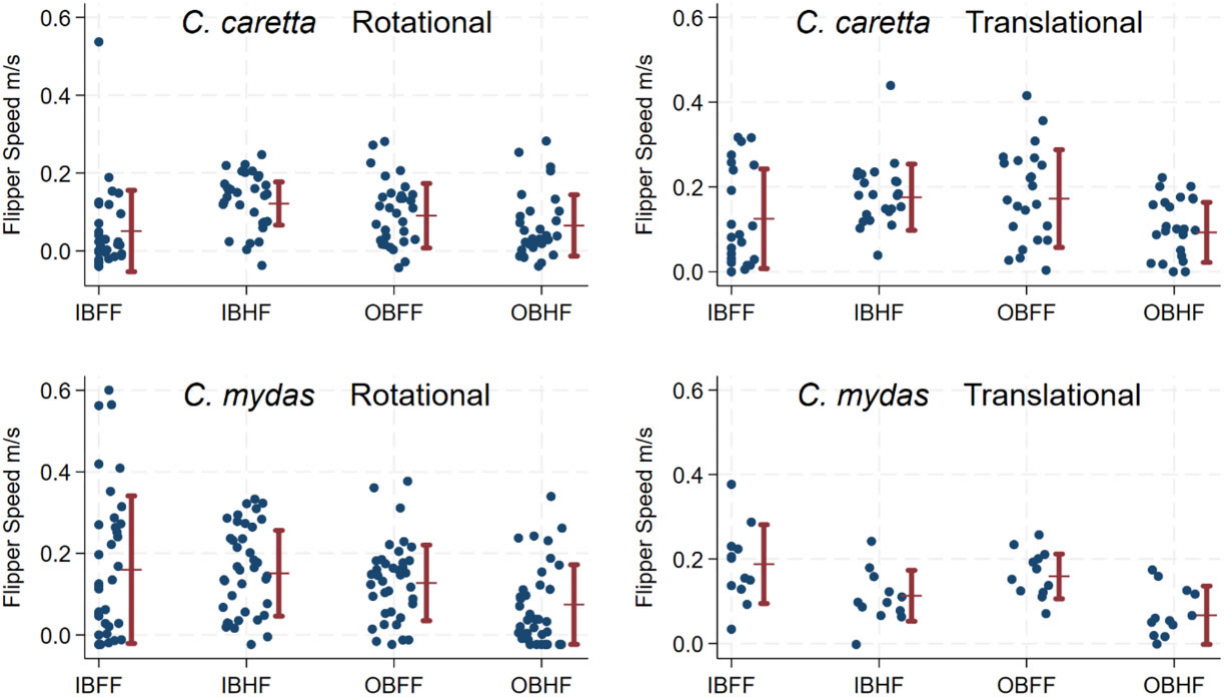
Means of flipper speeds (m s^−1^) (red) over raw flipper speed data (blue).

Mean stroke speeds are measured against raw data in Fig. 7. Data was not distributed normally (V = 15.712), so bootstrapped values were used. Speed percentages were made by dividing the average speed of the faster limb over the average speed of the slower limb, and then multiplying by 100. Regardless of the turn type, stroke speed of the forelimbs displayed no difference with the hindlimbs, but inboard limbs were 30% faster than outboard limbs (S1).

Looking at broad pairings of limbs, no difference was found between the stroke speeds of forelimbs and hindlimbs by *C. caretta*, and there was no difference between the stroke speeds of inboard and outboard limbs. Looking at specific pairings of limbs, outboard forelimbs were 62.5% faster than inboard forelimbs, whereas the inboard hindlimbs were 114.3% faster than outboard hindlimbs.

During rotational turns by *C. caretta*, no difference was apparent between the stroke speeds of forelimbs and hindlimbs. The outboard forelimbs were 80.0% faster than inboard forelimbs, whereas inboard hindlimbs were 57.1% faster than outboard hindlimbs. For translational turns by *C. caretta*, there was no difference between the stroke speeds of forelimbs and hindlimbs as well as the stroke speeds of inboard between outboard forelimbs. Regarding the stroke speeds of forelimb pair, outboard forelimbs and inboard forelimbs were not different, whereas for the hindlimb pair, the inboard hindlimbs were 100% faster than outboard hindlimbs.

*C. mydas* forelimb movements during turning were 36.4% faster than hindlimbs with inboard forelimbs faster than outboard limbs. Stroke speeds of the inboard hindlimbs were 100% faster than outboard hindlimbs. During rotational turns, there was no difference between forelimbs and hindlimbs with inboard limbs being 60% faster than outboard limbs. For translational turns, strokes by the forelimbs were 88.9% faster than hindlimbs, and inboard limbs were 36.4% faster than outboard limbs.

Statistics for stroke frequency comparisons are provided in S1. Data was not distributed normally (V = 12.312), so bootstrapped values were used instead. When considering the turn type, the stroke frequency of rotational turns of both sets of limbs were not different, but the outboard limbs stroked more frequently by 31.6% than inboard limbs. Comparing the stroke frequencies of forelimb pair, outboard forelimbs stroked 69.3% more frequently than inboard forelimbs. Inboard hindlimbs stroke frequency was not different from the outboard hindlimbs frequency. There was no difference between the stroke frequencies of forelimbs and hindlimbs during translational turns, but outboard limbs stroked 15.9% more frequently than inboard limbs. The stroke frequency of outboard forelimbs and inboard forelimbs showed no statistically significant difference, whereas the outboard hindlimbs (7.72 ± 0.74 Hz) stroked 31.5% more frequently than inboard hindlimbs.

Interaction models (statistics provided in S2) were used to investigate stroke speed, and stroke frequency. An interaction between species and turn type in predicting stroke speed was found. The reference category, or the constant, was *C. caretta*, performing a rotational turn. There was a main effect of species, with *C. mydas* producing faster strokes during rotational turns than *C. caretta*, and a main effect of turn type, with *C. caretta* producing faster strokes during translational turns than during rotational turns, and an interaction between species and turn type, meaning that the effect of turn type was lower for *C. mydas* than it was for *C. caretta*.

With stroke frequency, it was found that there was no interaction between species and turn type in predicting stroke frequency; the effect of turn type was not different for *C. mydas* than it was for *C. caretta*. There was no main effect of species, with *C. mydas* performing rotational turns no differently than *C. caretta*. There was, however, a significant main effect of turn type, with *C. caretta* having a greater stroke frequency during translational turns than during rotational turns.

Contingency table examination revealed a difference between species, regardless of the type of turn (Table 2). The inboard and outboard forelimbs of *C. caretta* were significantly positively correlated (*p* < 0.05), whereas in *C. mydas*, inboard and outboard forelimbs were not significantly correlated (*p* > 0.05). The difference between the co-occurrence of forelimbs in each species was investigated even further by constructing a z-test representing the species by synchronization interaction. The z-score was -2.618, and this z-score is significant (*p* < 0.05), indicating a difference in flipper usage between the species.

**Table 2:** Contingency table analysis, displaying species, outboard forelimbs, inboard forelimbs, and correlations

				Inboard				
				No Stroke	Stroke	Total	Proportion	Correlation
Species	*C. caretta*	Outboard	No Stroke	14	0	14	26.42	0.2889
			Stroke	29	10	39	73.58	
			Total	43	10	53	100	
	*C. mydas*	Outboard	No Stroke	4	5	9	17.65	−0.2182
			Stroke	30	12	42	82.35	
			Total	34	17	51	100	
	Total	Outboard	No Stroke	18	5	23		
			Stroke	59	22	81		
			Total	77	27	104		

Notes: There was a difference of differences in proportions (z=-2.618), and this is significant (*p* < 0.05). There was a difference between the two correlations, with the difference being 0.5071 (z = 2.684), and this is significant (*p* < 0.05).

## Discussion

### Species comparisons of turning performance

This study was an analysis and comparison of turning performance between two species of juvenile sea turtles: *C. caretta* and *C. mydas*. Predation by birds and fish is high for juveniles so an understanding of turning performance for predator avoidance is important. Turning performance was found to differ between the two species during rotational turns (Fig. 5). However, mean angular velocity of translational turns did not differ between species (Fig. 5). Although *C. caretta* had a higher maximum angular velocity for translational turns, the range of turning performance overlapped for the two species. The turtles displayed similar mean turn radii during mean translational turns and minimum rotational turns.

Stability is the quality of resisting forces that cause deviation in control or trajectory (Webb 2004). High stability is antithetical to high turning performance. Initially, it was hypothesized that juvenile *C. caretta* had reduced turning performances due to dorsally keeled ridges on their carapaces, which could increase stability. However, it was found that angular velocity differed between the two species during rotational turns. This could imply that there is a difference in stability between species during rotational turns. Stabilities are likely similar during translational turns, as the turning performances of the two species of sea turtles did not differ. The difference found between species during rotational turns differs from a previous study by Dougherty et al. (2010) comparing stability between hatchling *C. caretta* and *C. mydas*. He found that their stabilities did not significantly differ from each other, despite the presence of keeled ridges (Dougherty et al. 2010).

**Fig. 8:**
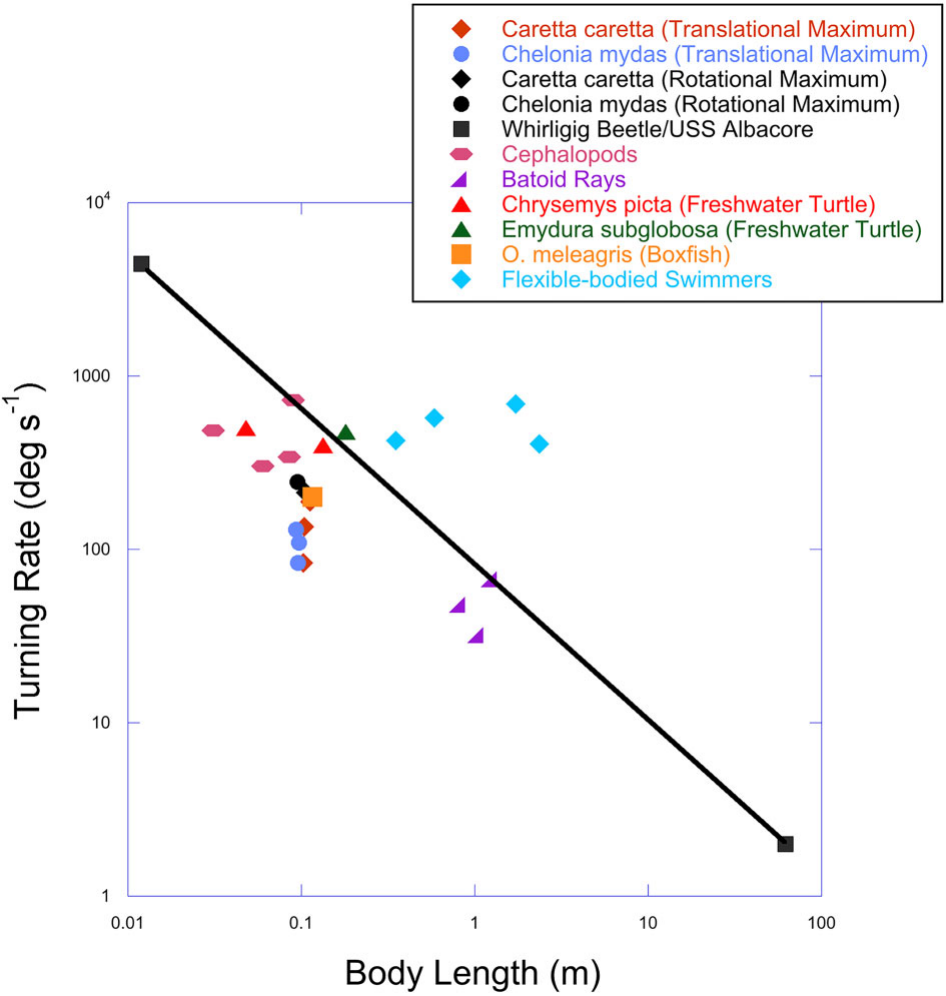
Comparison of turning rate (deg s^−1^) with respect to body length (meters). The line connects the smallest (whirligig beetle) and the largest (USS Albacore) rigid-bodied swimmers and functions to separate rigid bodies from flexible-bodied swimmers based on turning performance. Blue diamond symbols above the line represent flexible-bodied swimmers, while symbols below the line represent rigid-bodied swimmers. Data from Webb (1976, 1983), Hui (1985), Foyle and O’Dor (1988), Miller (1991), Blake et al. (1995), Gerstner (1999), Walker (2000) and Fish (1997, 2002).

Comparison of angular velocities of the sea turtles with other species that are flexible- and rigid-bodied swimmers showed the turning performance of sea turtles was similar to other rigid-bodied swimmers (Fig. 8; Hui 1985; Miller 1991; Blake et al. 1995; Largess and Mandelblatt 1999; Walker 2000; Fish 2002; Fish et al. 2003; Fish and Nicastro 2003; Rivera et al. 2006; Parson et al. 2011; Jastrebsky et al. 2016; Helmer et al. 2017; Fish et al. 2018; Mayerl et al. 2018). The turning performance in regard to angular velocity of the sea turtles fell below the line between the smallest (whirligig beetle, *Dineutes horni*) and largest (USS Albacore submarine, *AGSS-569*) rigid-bodied swimmers, respectively (Miller 1991; Fish and Nicastro 2003; Parson et al. 2011; Fish 2020; Downs et al. 2023). Sea turtle turning performance conforms with the other rigid-bodied swimmers. This supports the hypothesis that the rigid carapace of sea turtles constrains their ability to turn. The maximum turning rates of sea turtles were higher than that of batoid rays, comparable to the boxfish *Ostracion meleagris*, and lower than the angular velocity of cephalopods (Foyle and O’Dor 1988; Walker 2000; Parson et al. 2011; Jastrebsky et al. 2016; Fish et al. 2018). The turning rates of the sea turtles were lower compared to the freshwater turtles, *Chrysemys picta* and *Emydura subglobosa* (Rivera et al. 2006; Mayerl et al. 2018). While the juvenile sea turtles are comparable in size to adult *C. picta*, the sea turtle turning performances are lower than both juvenile and adult *C. picta*.

Rivera et al. (2006) stated that for a rigid-bodied swimmer to perform a turn, an asymmetry in forces between inboard and outboard control surfaces is required. As secondarily aquatic tetrapods, turtles are limited to two pairs of limbs and a tail as control surfaces (Mayerl et al. 2018). With most turtle species having reduced tails, they are limited to only their fore- and hindlimbs as control surfaces for propulsion and maneuvering. Freshwater turtles, whose fore- and hindlimbs still resemble terrestrial turtle limbs, use both the fore- and hindlimbs to turn in an aquatic environment. Rivera et al. (2006) found that painted turtles consistently performed turns by statically holding their abducted inboard forelimbs to turn, while rowing with the outboard forelimb and both hindlimbs.

The proportions in size between forelimbs and hindlimbs in sea turtles are different from that of freshwater turtles. Both *C. picta* and *E. subglobosa* have hindlimbs that are closer in length to their forelimbs (Mayerl et al. 2017), whereas sea turtles have enlarged and elongated forelimbs, and small paddle-like hindlimbs (Renous and Bels 1991; Rivera et al. 2011). Based on this, freshwater turtles would be expected to have higher turning performances because the force produced from forelimbs and hindlimbs would not be as disproportionate as it is in sea turtles. Control surfaces on the distal ends of the limbs differ, too, with the ratios of manus-pes area in sea turtles being larger (manus-pes ratio of 2.37) than that of freshwater turtles (manus-pes ratio of 0.5) (Mayerl et al. 2017).

### Sea turtle limb kinematic differences

**Fig. 9:**
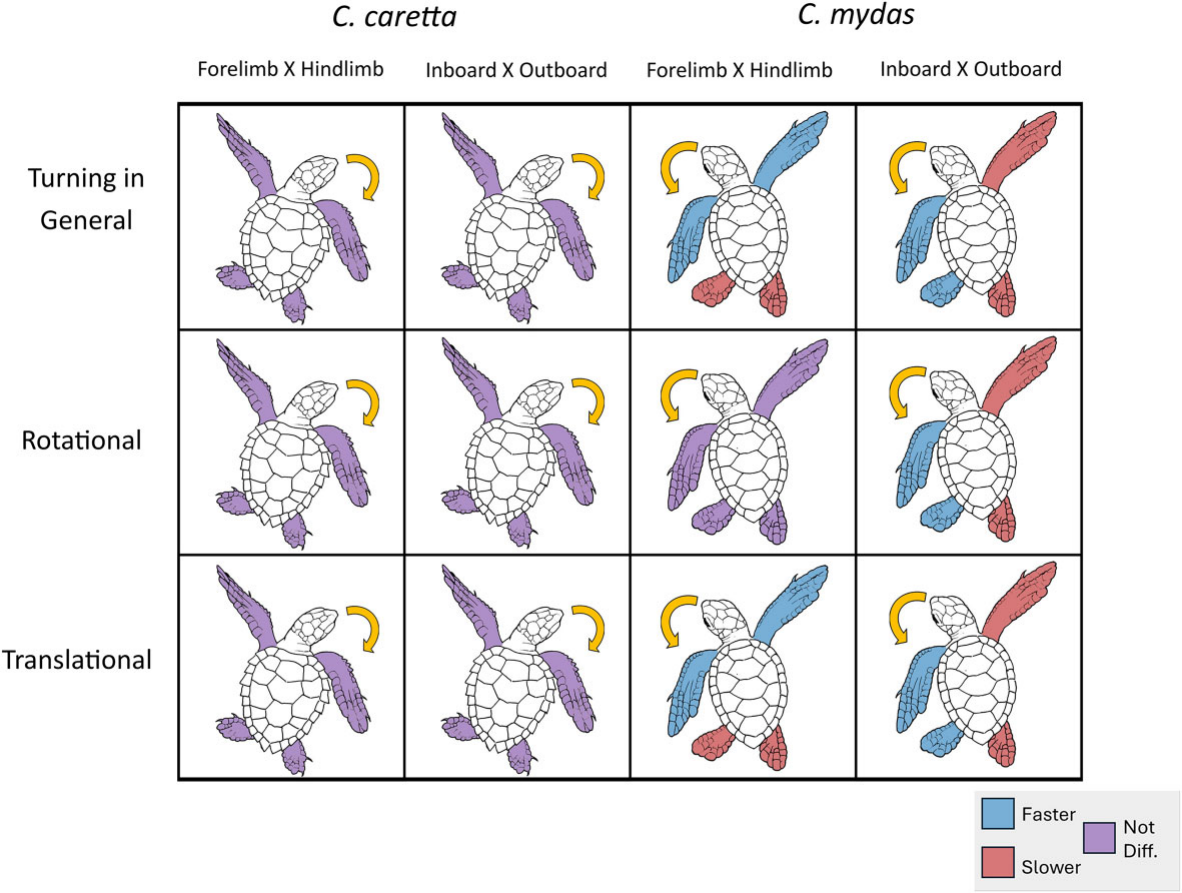
Differences in speed (m s^−1^) between broad limb pairings (Forelimbs vs Hindlimbs; Outboard Limbs vs Inboard Limbs). Within *C. mydas*, differences were consistently found within broad pairings: forelimbs were faster than hindlimbs, and inboard limbs were faster than outboard limbs, regardless of the turn type (rotational and translational). This might reflect the differences between their angular velocities during rotational and translational turns: the angular velocities of *C. caretta* showed no difference between rotational and translational turns, while the angular velocities of *C. mydas* differed between turn types.

**Fig. 10:**
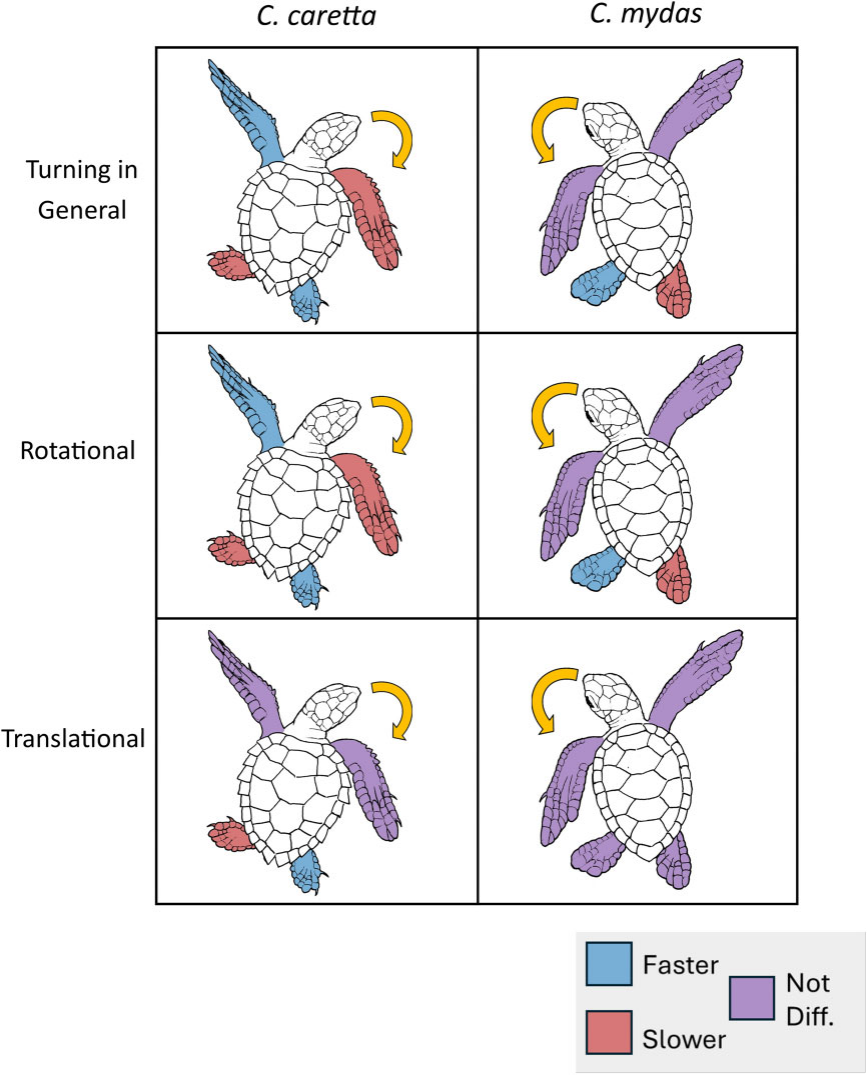
Differences in speed (m s^−1^) between specific limb pairings (Outboard Forelimb vs Inboard Forelimb; Outboard Hindlimb vs Inboard Hindlimb). Within *C. caretta*, differences in stroke speed were only found in specific pairs of limbs. In both rotational and translational turns, *C. caretta* was consistent in producing faster strokes from inboard hindlimbs. When disregarding turn type, the outboard forelimb was faster than the inboard forelimb. Only one specific pairing in *C. mydas* showed differences: during rotational turns, the speeds of inboard and outboard hindlimbs differed while the two forelimbs were similar; during translational turns, the forelimbs and hindlimbs were similar.

Stroke speeds were analyzed using linear mixed model regressions, comparing the stroke speeds of limb pairs, including broad pairings (forelimbs vs hindlimbs; inboard limbs vs outboard limbs) (Fig. 9), as well as specific pairings (inboard forelimbs vs outboard forelimbs; inboard hindlimbs vs outboard hindlimbs) (Fig. 10).

Differences in limb speed indirectly suggest differences in turning performance. The interaction model showed that both main effects differed between species in terms of limb speed: during rotational turns, *C. mydas* had faster forelimb strokes than *C. caretta; C. caretta* had faster forelimb strokes during translational turns than during rotational turns. The differences in limb speed between species during rotational turns, therefore indirectly supports the hypothesis that C. mydas performed faster turns. Additionally, the interaction between the main effects showed a difference between species in terms of their limb usage. Stroke speed in forelimbs changed less between rotational and translational turns for *C. mydas* than it did for *C. caretta*.

The analysis of the coordination of flippers revealed another difference between the two species of sea turtles, which pertains to each species’ use of forelimbs: In *C. caretta*, the two forelimbs were positively correlated but in *C. mydas*, the correlation between the two forelimbs was negative. The difference between the two correlations was significant. This seems to suggest that turns initiated by *C. caretta* more often started with the use of both forelimbs, while turns initiated by *C. mydas* more often started with the use of one forelimb. This analysis can perhaps be connected to the differences seen in stroke speed between flippers: in both species, the inboard hindlimbs were faster than the outboard hindlimbs, so the two species aren’t different in this regard; *C. caretta* had a faster outboard forelimb during turns in general, while the forelimbs of *C. mydas* did not differ during turns in general.

Forelimb stroke frequency is similar in the two species but differed between rotational and translational turn types. Forelimb stroke frequencies of both species were greater during translational turns than during rotational turns. The interaction model further confirms frequencies as being significantly different based on turn type, and not different based on species. Given that information, limb pair differences were investigated based on turn type, and not species. During both turn types, outboard limbs had a greater stroke frequency than inboard limbs. Only during rotational turns, however, do the stroke frequencies between forelimbs differ.

*C. caretta* hatchlings have been examined for their responses to wave-induced rotational displacement, including yaw rotational displacement (Avens et al. 2003). Although the study was not an examination of turning performance itself, it is similar for counter-rotation for positional control. The turtles stabilized themselves by extending the outboard hindlimb in relation to the rotational displacement, while using both forelimbs to paddle (Avens et al. 2003). Although hindlimbs were outboard in relation to the yaw displacement, hindlimbs were inboard in relation to the correction (Avens et al. 2003). The actions involved during the correction appear to be comparable to the actions of *C. caretta* in the current study; whereby, *C. caretta’s* inboard hindlimbs moved faster than their outboard hindlimbs during turns in general, and *C. caretta’s* inboard hindlimbs being significant in relation to angular velocity.

### Implications of a constrained body

As members of the two species examined grow, their morphologies change allometrically (Davenport and Clough 1986; Salmon and Scholl 2014), and in the case of *C. caretta*, the mechanics of their swimming changes as well (Davenport and Clough 1986). Changes in morphology and mechanics, as well as an increase in size, would likely result in differences in turning performance. The juvenile turtle’s overall turning performance would probably decrease, as predicted by previous studies showing turning performances in relation to body size (Webb 1976, 1983; Hui 1985; Foyle and O’Dor 1988; Miller 1991; Blake et al. 1995; Fish 1997, 2002; Gerstner 1999; Walker 2000).

As large, strong animals, and due to their imperiled species status, it would be difficult to use adult sea turtles in an experimental study of maneuverability. Data, however, can partially be acquired from observational studies based on chance video records in the wild. An aerial video showed *C. mydas* turning to dodge bites from a tiger shark (https://www.youtube.com/watch?v=4Y26l4J7Zmk). Another video titled *Turtle Fights Off Shark After Ocean Battle* (https://www.youtube.com/watch?v=aEvS2b1t_Vk), depicted a situation in which a large *C. caretta* eluded an attack from a tiger shark. The video showed the turtle performing both rotational and translational yaw turns, as well as rolls to avoid the attack. Large leatherback sea turtles (*Dermochelys coriacea*) similarly can be aggressive towards predators (Engbring et al. 1992). In the video *Extreme Free Dive Leatherback Turtle Attack* (https://www.youtube.com/watch?v=armYanU3wFc), the turtle demonstrates two rotational turns as a defensive tactic to face a possible threat.

## Conclusions

Juvenile sea turtles of the species *C. caretta* and *C. mydas* differed in turning performance during rotational turns but did not differ in turning performance or turning radii during translational turns. Juvenile sea turtles align with other rigid-bodied swimmers in that their turning performances are below that of flexible-bodied swimmers of similar sizes. Throughout both types of turns, the mechanics of *C. caretta* flippers remained mostly consistent. Yet, *C. mydas* flippers showed some variations, which may be reflected in the differences between their angular velocities during rotational and translational turns. Species differed in terms of their limb usage: stroke speed in limbs changed less between rotational and translational turns for *C. mydas* than it did for *C. caretta*. There are differences between species in how they use their forelimbs in turns. *C. caretta* tended to start turns with both forelimbs more often than *C. mydas*. This dichotomy likely reflects the differences in speeds found between limb pairs. Fore- and hindlimb stroke frequency in both species is greater during translational turns than during rotational turns. Despite constraints on sea turtles due to their body rigidity, the flexibility of their turning behaviors and ability to turn rapidly are ecologically important for defense to out-maneuver predators.

## Supplementary Material

obag017_Supplemental_Files

## Data Availability

Data are available from the West Chester University institutional data repository: http://digitalcommons.wcupa.edu/bio_data/12

## Notes

Summary Statement: Juvenile sea turtles use combinations of fore- and hindlimbs to perform translational and pure rotational turns in water.
