# Correction to: Aquatic Turning Performance in Juvenile Loggerhead and Green Sea Turtles

**DOI:** 10.1093/iob/obag027

**Published:** 2026-06-17

This is a **correction** to:

M T Wileyto, F E Fish, S E Trail, J Wyneken, R Kramer-Bottiglio, Aquatic Turning Performance in Juvenile Loggerhead and Green Sea Turtles, *Integrative Organismal Biology*, Volume 8, Issue 1, 2026, obag017, https://doi.org/10.1093/iob/obag017

In the originally published version of this manuscript, author, R Kramer-Bottiglio’s hyphenated name was inverted. This error has been corrected and subsequently RKB’s initials have also been updated throughout the paper.

Additionally, the ONR funding acknowledgments are incorrect and have been corrected as: N00014-21-1-2417 and N00014-24-1-2162.

