# Supplementary figures and images for: Aquatic Turning Performance in Juvenile Loggerhead and Green Sea Turtles
Source: Integr Org Biol. 2026 Apr 24;8(1):obag017. doi: 10.1093/iob/obag017 (PMC13168891; doi:10.1093/iob/obag017)

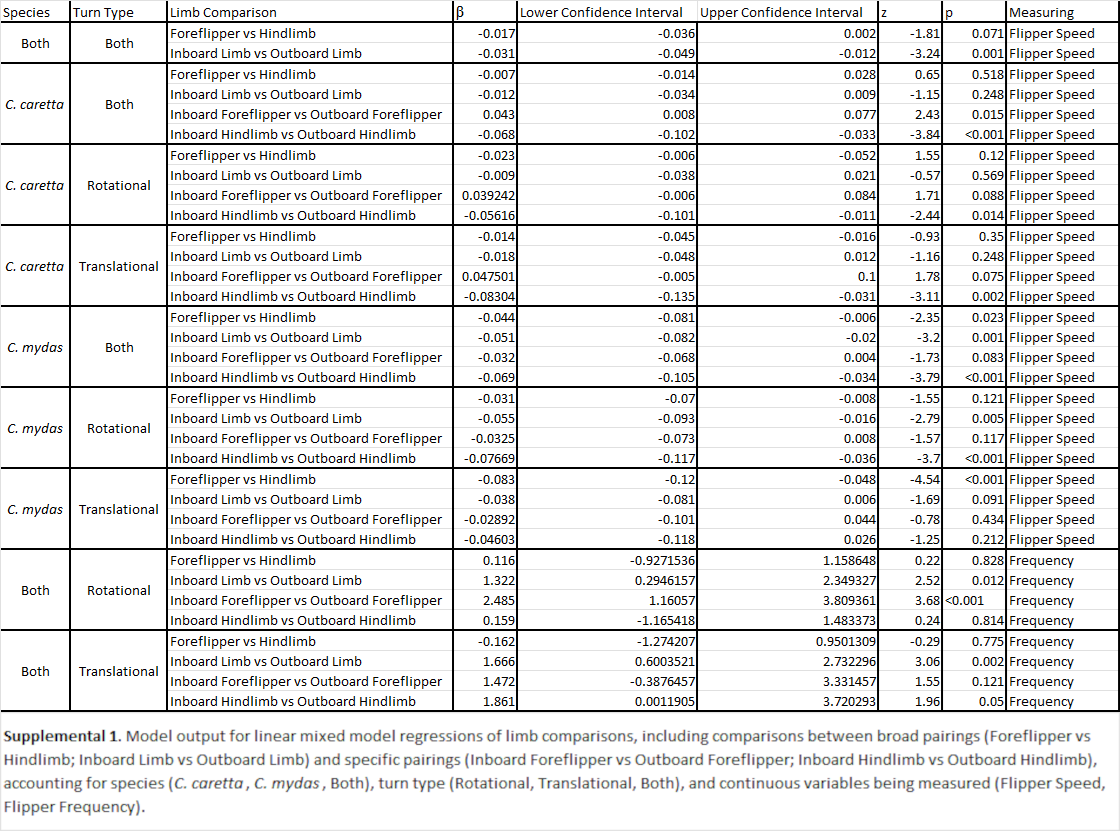

Supplement: obag017_Supplemental_Files [file obag017_supplemental_files.zip › S1.png]

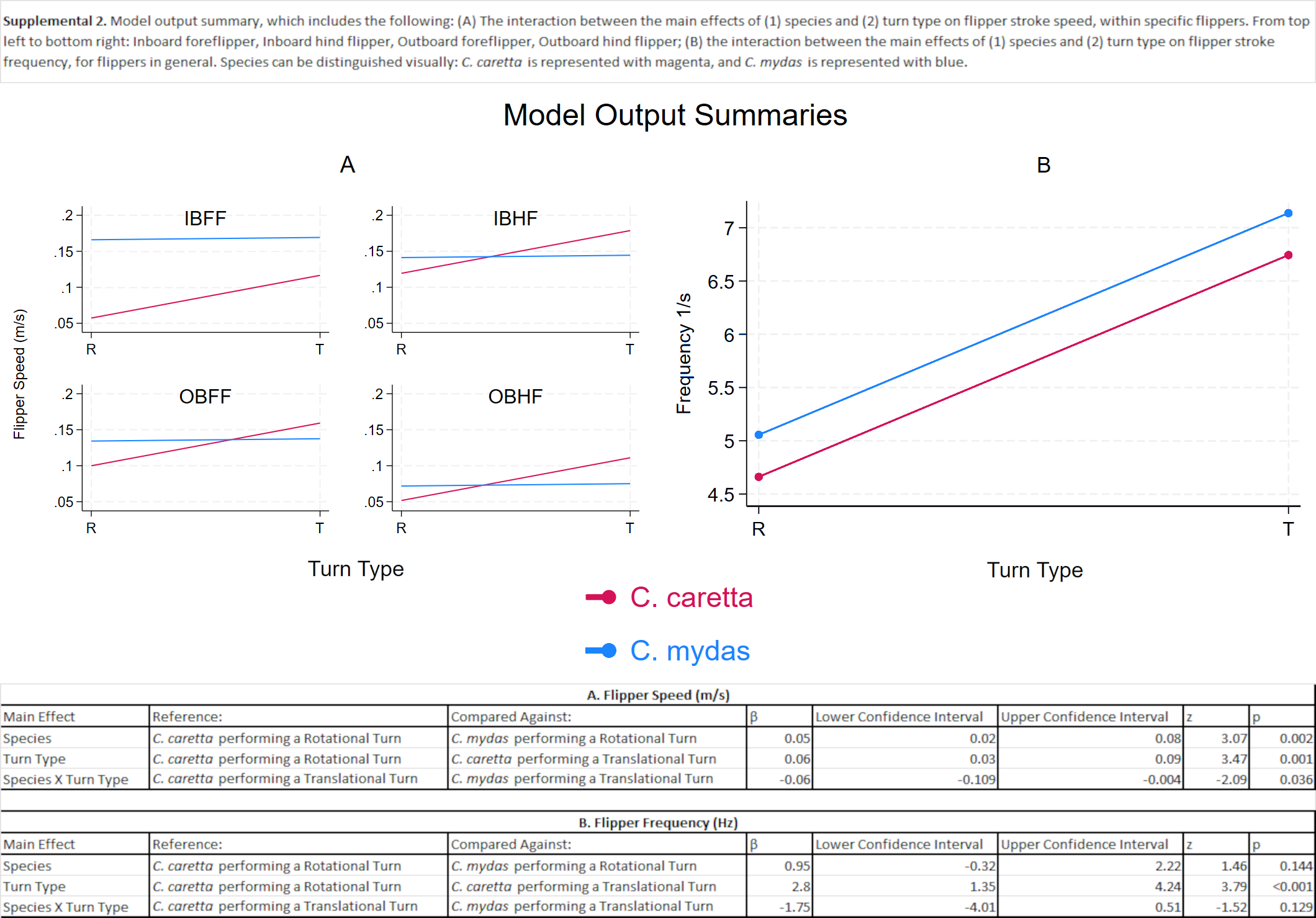

Supplement: obag017_Supplemental_Files [file obag017_supplemental_files.zip › S2.png]

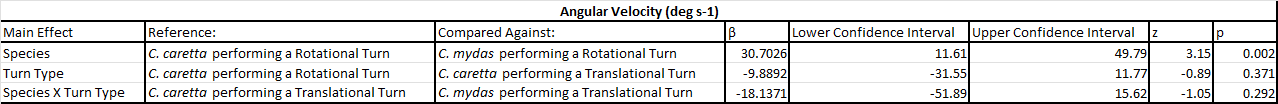

Supplement: obag017_Supplemental_Files [file obag017_supplemental_files.zip › S3_Table.png]
